# Wnt5a Signals through DVL1 to Repress Ribosomal DNA Transcription by RNA Polymerase I

**DOI:** 10.1371/journal.pgen.1006217

**Published:** 2016-08-08

**Authors:** Randall A. Dass, Aishe A. Sarshad, Brittany B. Carson, Jennifer M. Feenstra, Amanpreet Kaur, Ales Obrdlik, Matthew M. Parks, Varsha Prakash, Damon K. Love, Kristian Pietras, Rosa Serra, Scott C. Blanchard, Piergiorgio Percipalle, Anthony M. C. Brown, C. Theresa Vincent

**Affiliations:** 1 Department of Cell and Developmental Biology, Weill Cornell Medical College, New York, New York, United States of America; 2 Department of Physiology and Biophysics, Weill Cornell Medical College, New York, New York, United States of America; 3 Department of Cell and Molecular Biology, Karolinska Institute, Stockholm, Sweden; 4 Biology Program, New York University Abu Dhabi, Abu Dhabi, United Arab Emirates; 5 Department of Physiology and Pharmacology, Karolinska Institute, Stockholm, Sweden; 6 Department of Laboratory Medicine, Center for Molecular Pathology, Lund University, Lund, Sweden; 7 Department of Cell, Developmental, and Integrative Biology, University of Alabama at Birmingham, Birmingham, Alabama, United States of America; 8 Tri-Institutional PhD program in Chemical Biology, Weill Cornell Medical College, New York, New York, United States of America; DKFZ, GERMANY

## Abstract

Ribosome biogenesis is essential for cell growth and proliferation and is commonly elevated in cancer. Accordingly, numerous oncogene and tumor suppressor signaling pathways target rRNA synthesis. In breast cancer, non-canonical Wnt signaling by Wnt5a has been reported to antagonize tumor growth. Here, we show that Wnt5a rapidly represses rDNA gene transcription in breast cancer cells and generates a chromatin state with reduced transcription of rDNA by RNA polymerase I (Pol I). These effects were specifically dependent on Dishevelled1 (DVL1), which accumulates in nucleolar organizer regions (NORs) and binds to rDNA regions of the chromosome. Upon DVL1 binding, the Pol I transcription activator and deacetylase Sirtuin 7 (SIRT7) releases from rDNA loci, concomitant with disassembly of Pol I transcription machinery at the rDNA promoter. These findings reveal that Wnt5a signals through DVL1 to suppress rRNA transcription. This provides a novel mechanism for how Wnt5a exerts tumor suppressive effects and why disruption of Wnt5a signaling enhances mammary tumor growth *in vivo*.

## Author Summary

Synthesis of the translation machinery, including the mega-Dalton, RNA-protein ribosome complex, serves as a key driver of cellular growth and proliferation. It is therefore unsurprising that ribosomal biogenesis is under intricate regulation. The process through which ribosomes are made entails the coordination of components from diverse signaling pathways in both normal and diseased cells. Both oncogenes and tumor suppressors can influence this orchestration by impinging upon the rate-determining steps of RNA Polymerase I-mediated transcription of ribosomal RNA (rRNA) and the coupled process of ribosome assembly. In this study we investigated whether the secreted protein Wnt5a, an antagonist of mammary tumor growth, regulates rRNA synthesis in breast cancer cells. We find that the induction of Wnt5a signaling disturbs assembly of the RNA polymerase I machinery, leading to a repressive rDNA chromatin state that is not amenable to active rRNA gene transcription. Wnt5a signaling represses rRNA synthesis by stimulating nucleolar accumulation of Dishevelled1 (DVL1), a downstream effector of Wnt5a signaling, while having no such effect on DVL2 or DVL3. Wnt5a-induced accumulation of DVL1 in the nucleolus directly interferes with the synthesis of rRNA, suggesting that a tumor suppressive effect of Wnt5a in breast cancer cells is mediated by DVL1-dependent repression of rRNA synthesis.

## Introduction

Cellular growth and proliferation depend critically on ribosome biogenesis and protein synthesis. The synthesis of ribosomes is initiated in the nucleolus through a complex, highly coordinated process that engages roughly 60–80% of the cell’s metabolic energy [1–4]. The transcription of rRNA is carried out by RNA Polymerase I (RNA Pol I), which transcribes 28S, 5.8S and 18S rDNA as a single, 47S precursor (pre)-rRNA. RNA Pol I does so by working in concert with auxiliary proteins to form a transcription-competent complex. Upstream binding factor (UBF) serves as a key component of the RNA Pol I machinery that aids its recruitment to the promoter region of the rDNA gene cassette [3–8]. The deacetylase SIRT7 facilitates this recruitment step through the de-acetylation of PAF53 [9–13]. SIRT7’s association with RNA Pol I at the rDNA transcription unit is therefore crucial to the regulation of rRNA synthesis. Correspondingly, SIRT7 regulation serves as an important means for modulating ribosome biogenesis and cellular proliferation during normal cell growth and in disease states, including cancer [14–16].

Wnt proteins are secreted signaling factors that regulate many physiological processes at the cell and tissue level [17,18]. Intracellular Wnt signaling is typically mediated through multi-functional Dishevelled proteins (DVLs), which serve as signaling hubs in multiple subcellular locations [18–21]. Misregulation of Wnt signaling, especially the canonical Wnt/β-catenin pathway, is often associated with oncogenesis [17,18]. Wnt5a signaling plays a less well-characterized role in tumor development but is understood to act via non-canonical, β-catenin-independent, Wnt signaling pathways [22,23]. In breast cancer, Wnt5a signaling has been shown to exert tumor suppressive effects and loss of Wnt5a expression has been associated with accelerated tumor growth [23–25]. However, the physiologically and pathologically relevant targets in breast cancer downstream of non-canonical Wnt signaling have yet to be fully elucidated.

It has been demonstrated previously that numerous growth and oncogenic signals, including epidermal growth factor (EGF) and RAS, directly increase rDNA transcription as means of promoting cellular proliferation [26]. We therefore hypothesized that counter-balancing cellular signals may decrease rDNA transcription in order to maintain homeostasis, and that Wnt5a may exert suppressive effects on tumor cell growth and proliferation through such means. In order to test this hypothesis, we set out to examine whether exogenous Wnt5a could impact tumor proliferation in breast cancer cells through the regulation of rDNA transcription.

Here, we demonstrate that Wnt5a represses rRNA synthesis in breast cancer cells by promoting nucleolar localization of DVL1 and its subsequent association with rDNA chromatin. Localization of DVL1 to rDNA gene cassette regions leads to a displacement of SIRT7 from the Pol I transcription machinery. Hence, Wnt5a signals through DVL1 to negatively regulate rDNA transcription. We substantiated this conclusion by showing that Wnt5a-null mammary tumor cells display enlarged nucleoli, increased proliferation and increased SIRT7 expression *in vivo*. Given that nucleolar size and elevated levels of rRNA synthesis correlate with poor prognosis in breast cancer, we posit that non-canonical signaling of Wnt5a through DVL1 and the consequent reduction in rRNA synthesis contributes to the role of Wnt5a in tumor suppression [24–26].

## Results

### Wnt5a signaling decreases rDNA transcription

**Fig 1:**
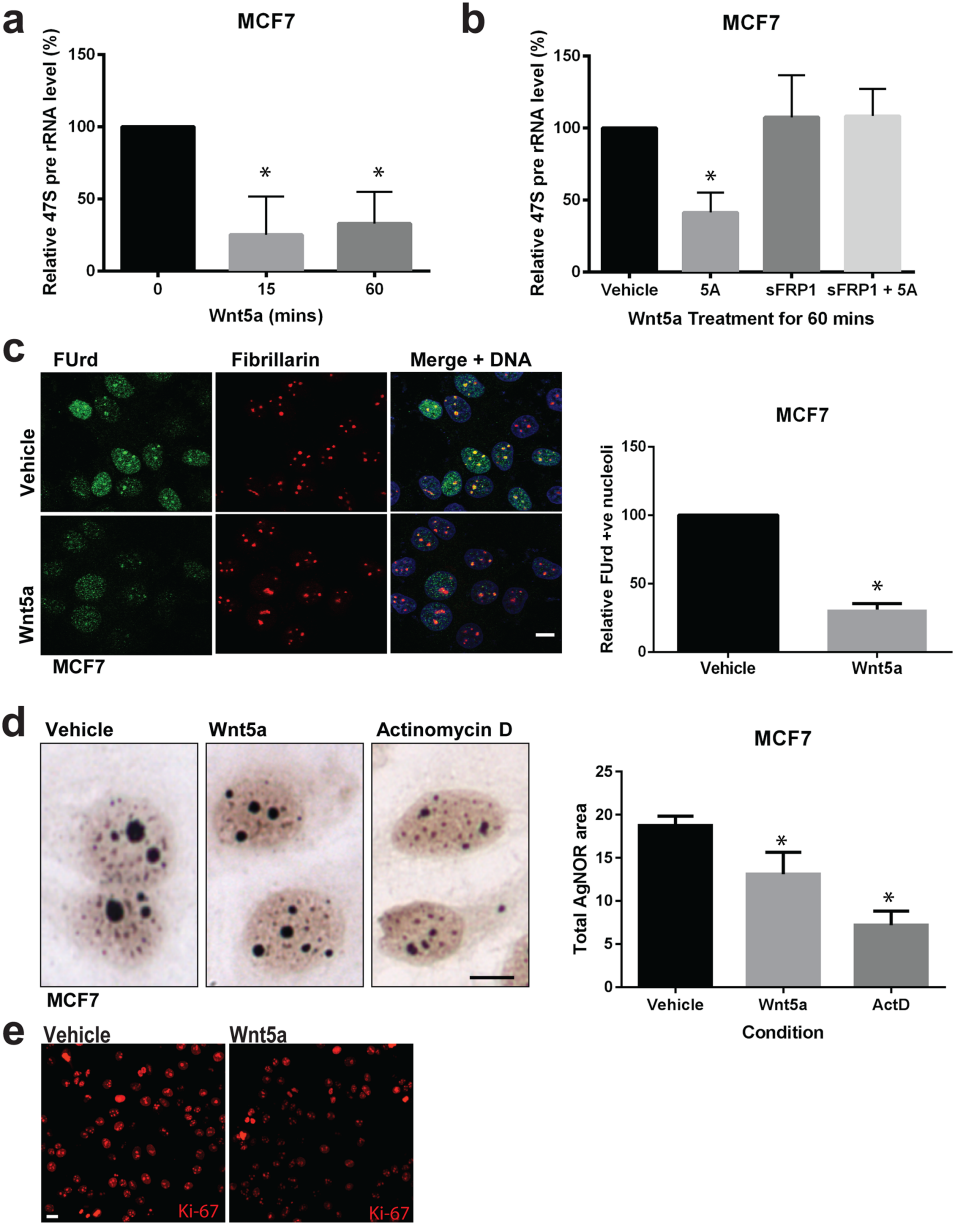
Wnt5a inhibits rRNA gene transcription and reduces the size of nucleoli. **(a)** Semi-quantitative RT-PCR analysis of 47S pre-rRNA transcript levels in MCF7 cells treated with Wnt5a for the indicated times. Error bars indicate ± SD. *P < 0.05 (n = 3). **(b)** The inhibitory effect of Wnt5a treatment for 60 mins on 47S rRNA synthesis is prevented by pre-treatment (60 mins) with sFRP1 in MCF7 cells. *P < 0.01 (n = 3). **(c)** *In situ* nuclear run-on assay of 5-Fluorouridine (FUrd) incorporation into nascent transcripts. MCF7 cells were treated with Wnt5a for 20 min and FUrd was detected by immunofluorescence (green). Cells were co-stained for Fibrillarin (red) and DNA (blue) to highlight nucleolar incorporation of FUrd (yellow in merged image). Scale bar = 10 μm. Quantitation of nuclei with bright nucleolar incorporation of FUrd in vehicle- and Wnt5a-treated cells. Error bars indicate ± SD. *P < 0.0001 (n = 3). **(d)** Silver staining of nucleolar organizer regions (AgNOR) of MCF7 cells treated with vehicle, 200 ng/mL Wnt5a, or 1 μg/mL Actinomycin D (ActD) for 4 hours. Scale bar = 10 μm. Quantification of AgNOR staining showing that Wnt5a and ActD both reduce the area of nucleoli. Error bars indicate ± SD *P <0.05; (n = 3). **(e)** Confocal immunofluorescence of Ki-67 (red) in MCF7 cells treated with either vehicle or with 200 ng/mL Wnt5a for 4 hours. Scale bar = 10 μm (n = 3).

The primary transcript of rRNA genes is a 47S pre-rRNA pre-cursor, which is rapidly processed into smaller, mature rRNA molecules that comprise key structural and functional elements of the large and small subunits of the ribosome [1,3]. Given the short half-life of the 47S pre-rRNA, its abundance serves as a marker of active rDNA transcription [27,28]. We therefore assayed rDNA transcription after treatment with recombinant Wnt5a protein by measuring the levels of 47S pre-rRNA, using human estrogen receptor positive (ER+) breast cancer cells (MCF7). Strikingly, Wnt5a treatment reduced 47S pre-rRNA by ≥50% within 15 min, demonstrating that Wnt5a acts to rapidly inhibit rRNA synthesis (Fig 1a). This effect was abrogated by the soluble Wnt antagonist sFRP1, confirming the specificity of Wnt pathway signaling [29,30] (Fig 1b). Consistent with Wnt5a signaling through a β-catenin-independent Wnt pathway, Wnt5a failed to induce accumulation of nuclear β-catenin (S1a Fig) but modestly induced DVL2 phosphorylation (S1b Fig) [22,23,31].

Given these observations we hypothesized that Wnt5a may signal through a non-canonical Wnt pathway to inhibit rRNA synthesis. To test this hypothesis, we performed *in situ* nuclear run-on assays in which synthesis of nascent RNA transcripts was monitored by incorporation of 5-Fluorouridine (FUrd) [32]. Using this assay, we observed that a 15 min treatment with Wnt5a produced a >60% drop in the proportion of cells exhibiting nucleolar FUrd (as defined by co-localization with the nucleolar marker Fibrillarin) suggesting that Wnt5a represses rDNA transcription (Fig 1c).

As the size of nucleolus typically reflects levels of rDNA transcription, we next asked whether Wnt5a treatment lead to changes in the nucleolar area as detected by AgNOR silver staining [26,33–35]. As a positive control, we treated cells with Actinomycin D (ActD), a potent inhibitor of transcription [36]. As expected, ActD treatment caused a substantial reduction in the total area of the nucleoli after 4 hours (Fig 1d). Wnt5a induced a significant decrease in the nucleolar area within the same time frame (Fig 1d). The relative reduction in nucleolar area mediated by Wnt5a was even more pronounced in the triple negative human breast cancer cell line (TNBC), BT549 (S1c Fig), indicating that these effects of Wnt5a are found in other breast cancer cell lines. Examination of the proliferation marker Ki-67 also revealed that Wnt5a treatment reduced cellular proliferation (Fig 1e). Moreover, BT549 cells constitutively expressing exogenous Wnt5a displayed smaller nucleoli and slower proliferation than control cells, as measured by MTT assay (S1d and S1e Fig). Reduced nucleolar areas were also observed in MCF7 expressing exogenous Wnt5a (S1d Fig). These data argue that Wnt5a signaling has a repressive effect on rRNA synthesis that restrains proliferation in breast cancer cells.

### Wnt5a signaling promotes DVL1 localization to nucleoli and rDNA chromatin

**Fig 2:**
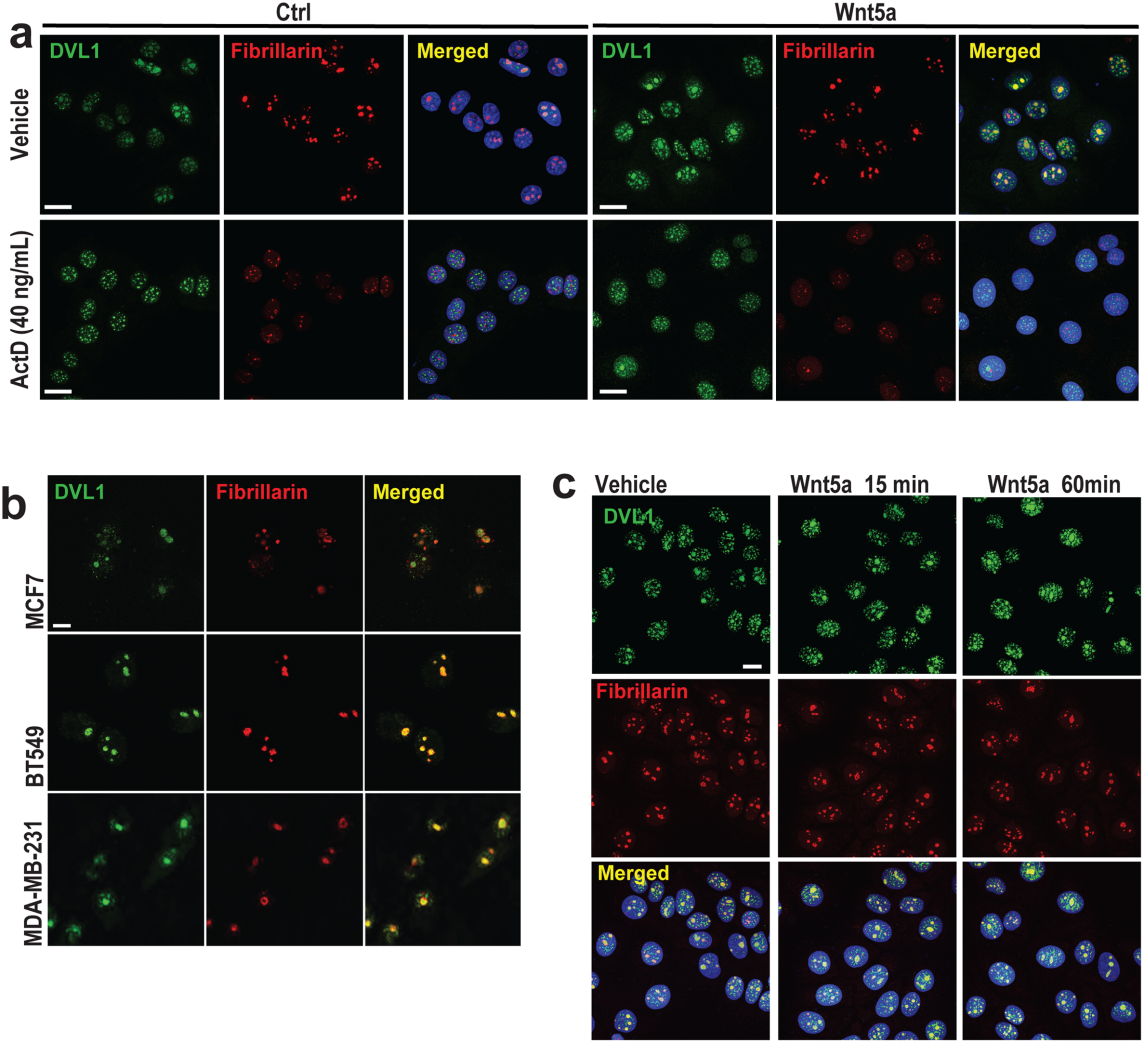
DVL1 accumulates in the nucleolus upon Wnt5a signaling. **(a)** Immunofluorescence and confocal microscopy using antibodies to DVL1 (green) and Fibrillarin (red) merged with DNA (blue) in MCF7 cells and MCF7 cells stably expressing Wnt5a treated with vehicle or with ActD, 40 ng/mL for 4 hours. Scale bar, 10μm (n = 3). **(b)** Co-localization (yellow) of DVL1 (green) and Fibrillarin (red) indicates that DVL1 is present in nucleoli of human breast cell lines MCF7, BT549 and MDA-MB-231. Scale bar, 10μm (n = 3). **(c)** Immunofluorescence of DVL1 (green), Fibrillarin (red) with DNA stain (blue) in MCF7 cells shows increased accumulation of DVL1 in nucleoli within 15 to 60 min of treatment with 200 ng/mL Wnt5a treatment. Scale bar = 10 μm (n = 3).

To investigate intracellular signaling effects of Wnt5a signaling, we next examined the subcellular distributions of endogenous DVL1, 2 and 3 proteins, in the presence and absence of exogenous Wnt5a expression in MCF7 cells. In control cells, DVL1 exhibited distinctive nuclear and sub-nuclear distributions, and co-localized with Fibrillarin (Fig 2a). In contrast, DVL2 and DVL3 were preferentially cytoplasmic and excluded from nucleoli as determined by the absence of co-localization with the nucleolar markers Fibrillarin and UBF (S2b Fig). Surprisingly, ActD treatment led to reduced distribution of DVL1 inside nucleoli in both control cells and cells overexpressing Wnt5a (Fig 2a) as has been shown previously for both Fibrillarin and UBF [36]. By contrast, the subcellular localization of DVL2 and DVL3 did not change upon ActD treatment (S2b Fig), suggesting a specific role for DVL1 in regulation of rDNA transcription. The nucleolar localization of DVL1 was further confirmed in three distinct breast cell lines (Fig 2b) and by using an alternative DVL1 antibody, which also revealed some cytoplasmic staining (S3a Fig). Evidence that DVL1 can be specifically localized to the nucleolus was further shown by ectopic expression of FLAG-tagged DVL1 in fibroblasts lacking endogenous DVL1 protein (S3b Fig) as well as by immuno-electron microscopy of MCF7 cells stably expressing Wnt5a, and of MDA-MB-231 breast cancer cells (S3c Fig). Given that the nucleolar localization of DVL1 was more prominent in cells stably expressing Wnt5a (Fig 2a, right hand panel), we next asked whether acute exposure to exogenous Wnt5a protein affects the cellular distribution of DVL1. We observed that the treatment of MCF7 cells with recombinant Wnt5a resulted in more prominent nucleolar staining of DVL1 at both 15 and 60 minute time points (Fig 2c). Taken together, these observations suggest that DVL1 is actively recruited into the nucleolus in response to Wnt5a signaling.

**Fig 3:**
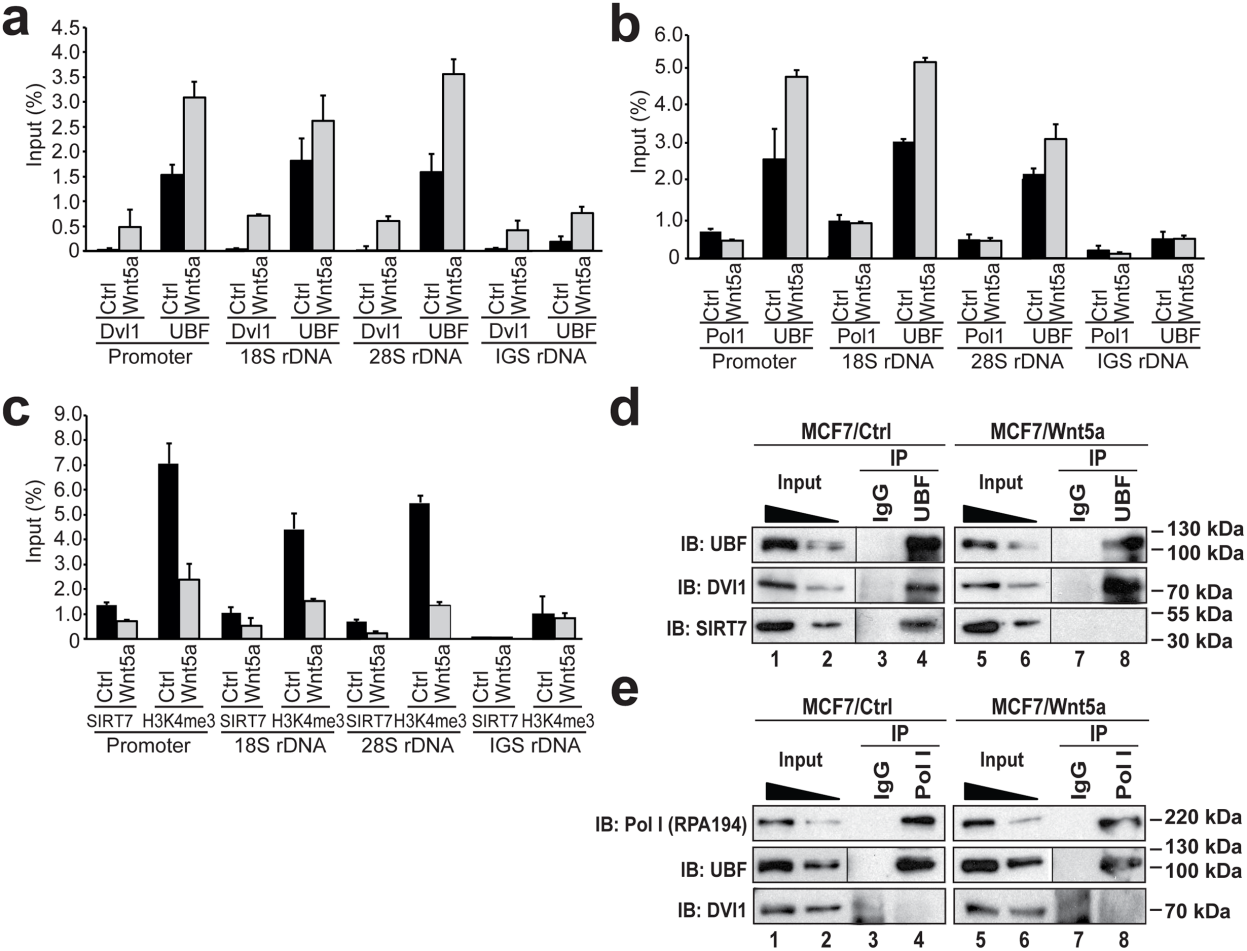
DVL1 is recruited to the rRNA gene cassette upon Wnt5a signaling, while SIRT7 levels are reduced. **(a)** Chromatin immunoprecipitation (ChIP) and qPCR analysis of DVL1 and UBF on rDNA chromatin isolated from MCF7 cells (Ctrl) and MCF7 cells stably expressing Wnt5a (Wnt5a). The rRNA gene promoter, 18S rDNA and 28S rDNA repeat, and IGSs were analyzed separately using antibodies against DVL1 and UBF (as indicated below the bar graphs). Values are presented as the percentage of input signal measured for each primer pair. Error bars indicate ± SD. (n = 3). **(b)** Equivalent ChIP and qPCR analysis of Pol I and UBF on chromatin isolated from the same cells. Error bars indicate ± SD (n = 3). **(c)** Equivalent ChIP and qPCR analysis of SIRT7 and H3K4me3 on chromatin isolated from the same cells. Error bars indicate ± SD. (n = 3). **(d)** Co-immunoprecipitation of DVL1 and SIRT7 with UBF from nuclear protein extracts of MCF7 cells (MCF7/Ctrl) or MCF7 cells stably expressing Wnt5a (MCF7/Wnt5a). Bound proteins were detected on immunoblots with antibodies against UBF, DVL1 and SIRT7. 12% of the input material is shown in Lanes 1 and 5, and 6% in Lanes 2 and 6. IP, immunoprecipitation; IB, immunoblotting. **(e)** Co-immunoprecipitation of DVL1 and UBF with Pol I from nuclear protein extracts of MCF7 cells (MCF7/Ctrl) or MCF7 cells stably expressing Wnt5a (MCF7/Wnt5a). Bound proteins were detected on immunoblots with antibodies against UBF and DVL1. Approximately 12% and 6% of the input material is shown in Lanes 1 and 5, and 2 and 6, respectively. IP, immuno-precipitation; IB, immuno-blotting (n = 3).

These observations led us to hypothesize that Wnt5a- mediated accumulation of nucleolar DVL1 might be directly involved in the inhibition of rRNA synthesis. To test this hypothesis, chromatin immuno-precipitation (ChIP) assays were performed on control MCF7 cells, and MCF7 cells stably expressing Wnt5a, to assess the occupancy of Pol I, and its specific regulators UBF and the de-acetylase SIRT7, on rDNA regions (S3d Fig) DVL1 occupancy on the same rDNA regions was also assessed. Consistent with the hypothesis, MCF7 cells stably expressing Wnt5a showed an increase in DVL1 and UBF localization to the rDNA promoter region as well as 18S and 28S regions and, to a lesser extent, inter-genic sequences (IGSs) (Fig 3a). By contrast, relatively modest changes in Pol I occupancy at rDNA regions were detected, particularly outside of the promoter region (Fig 3b). These observations either suggest that the increase in UBF association influences Pol I dissociation in a manner that masks the expected signal or that Wnt5a’s impact is to reduce the transcriptional competency of Pol I while its occupancy is unaffected. However, in cells expressing Wnt5a, SIRT7 occupancy was reduced at the rDNA repeat (Fig 3c). As observed in cells repressed for Pol I transcription, Wnt5a signaling increased UBF occupancy on all regions of the rDNA repeat that were examined (Fig 3a and 3b) [37]. Importantly, these effects also correlated with a reduction of histone 3 lysine 4 tri-methylation (H3K4me3), an epigenetic mark of transcriptionally active chromatin (Fig 3c)[37,38].

To rationalize DVL1’s apparent distribution throughout the rDNA repeat, we hypothesized that Wnt5a signaling may induce DVL1 to associate with either UBF or Pol I. Consistent with this model, immunoprecipitation assays performed on total nuclear lysates showed that DVL1 co-precipitated with UBF more efficiently in Wnt5a-expressing cells (Fig 3d, compare lanes 4 and 8). By contrast, DVL1 did not co-precipitate with Pol I, irrespective of Wnt5a expression (Fig 3e, compare lanes 4 and 8). Conversely, we observed that SIRT7 co-precipitated with UBF, and that this association was ablated upon Wnt5a expression (Fig 3d, compare lanes 4 and 8). These data argue that Wnt5a signaling enhances interactions between UBF and DVL1 and that nucleolar localization of DVL1 regulates rRNA synthesis by directly or indirectly inducing a loss of SIRT7 from the Pol I transcriptional machinery.

### DVL1 is required for Wnt5a-mediated inhibition of rDNA transcription

**Fig 4:**
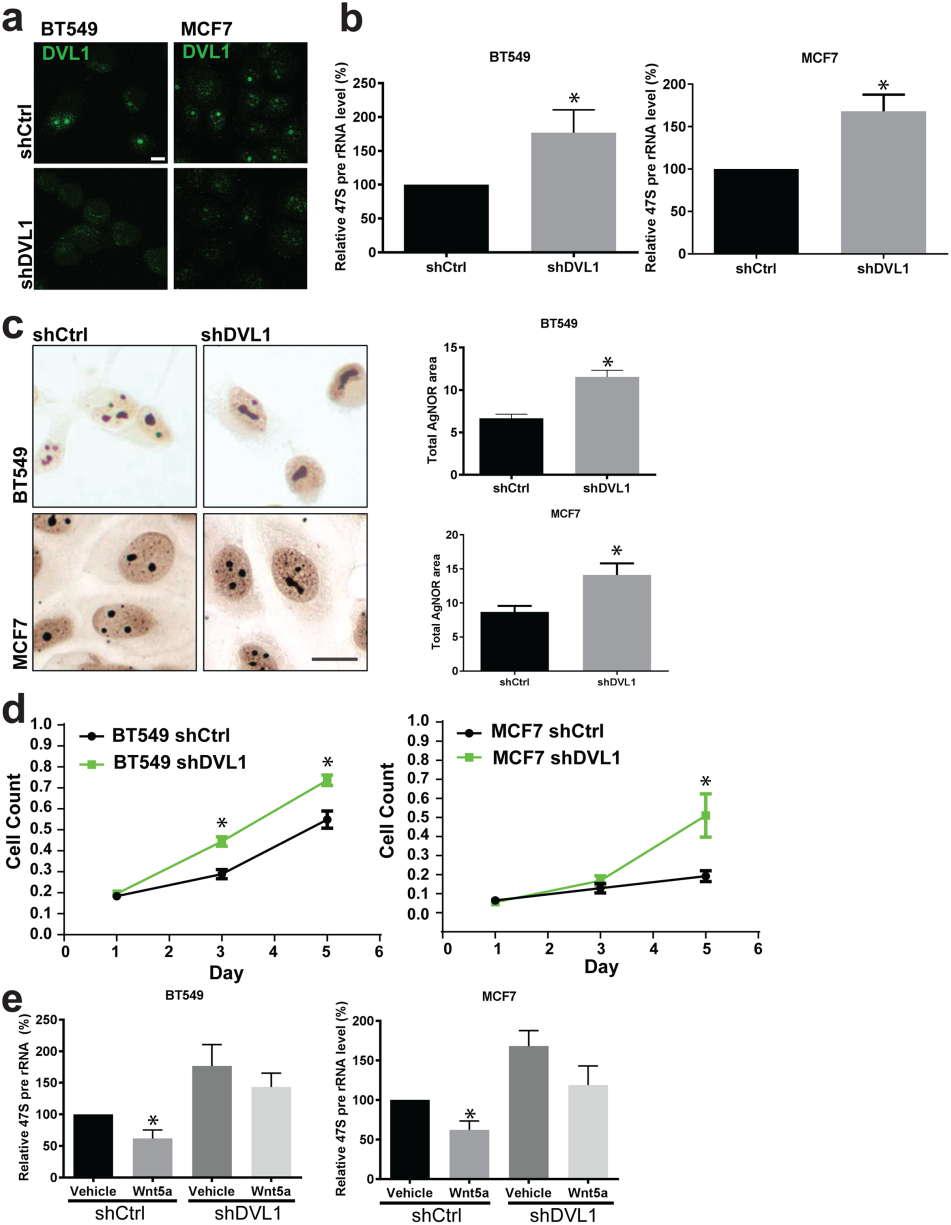
DVL1 is required for the reduction of rRNA synthesis mediated by Wnt5a. **(a)** Immunofluorescence showing reduced nucleolar staining of DVL1 (green) in BT549 and MCF7 cells transduced with a DVL1 shRNA vector (shDVL1), compared to non-silencing control (shCtrl) Scale bar = 10 μm (n = 3). **(b)** Knock-down of DVL1 in MCF7 and BT549 cells results in elevated 47S pre-rRNA transcript levels. Relative 47S RNA levels were measured by RT-PCR in shCtrl and shDVL1 cells. Error bars indicate ± SD. *P < 0.01 (MCF7) (n = 3). **(c)** AgNOR staining of BT549 and MCF7 cells transduced with shDVL1 and shCtrl. Scale bar = 10 μm. (n = 3). Quantification of AgNOR staining showing that silencing of DVL1 increases the mean area of nucleoli in both BT549 and MCF7 cells. Image J software was used to compare the total area of AgNOR staining in fields containing equivalent numbers of cells. Error bars indicate ± SD. *P < 0.05 (n = 3). **(d)** Proliferation assay shows that MCF7 and BT549 cells proliferate more rapidly when subjected to DVL1 gene silencing by shRNA. Data points were obtained in triplicate (n = 3). **(e)** Upon knock-down of DVL1, BT549 and MCF7 cells are refractory to the inhibitory effect of Wnt5a on rRNA synthesis. Relative 47S RNA levels were measured in shCtrl cells and shDVL1 cells with and without treatment of Wnt5a for 15 min. Error bars indicate ± SD. *P < 0.05 (n = 3).

We next asked whether DVL1 is an obligate downstream effector of Wnt5a-mediated suppression of rRNA synthesis. To address this question, we generated MCF7 and BT549 cells in which DVL1 expression was reduced by the stable expression of shRNAs specifically targeting DVL1 (Fig 4a), without altering levels of DVL2 and DVL3 (S4a Fig). In both cell lines, the reduction of DVL1 expression resulted in an increase in the steady-state levels of 47S pre-rRNA and enlargements in nucleolar size (Fig 4b and 4c), as well as an increased proliferation rate (Fig 4d). Similar results were obtained when siRNA oligonucleotides were used to reduce DVL1 expression in MCF7 cells (S4b Fig). Importantly, while Wnt5a treatment of control shRNA cells inhibited 47S pre-rRNA synthesis (as in wild-type cells), Wnt5a treatment failed to significantly reduce 47S pre-rRNA levels in DVL1 shRNA cells (Fig 4e). These results demonstrate a requirement for DVL1 in the Wnt5a-induced suppression of rDNA transcription and suggest that nucleolar DVL1 acts as an inhibitor of RNA Pol I-mediated transcription.

### Wnt5a signaling modulates rDNA transcription *in vivo*

**Fig 5:**
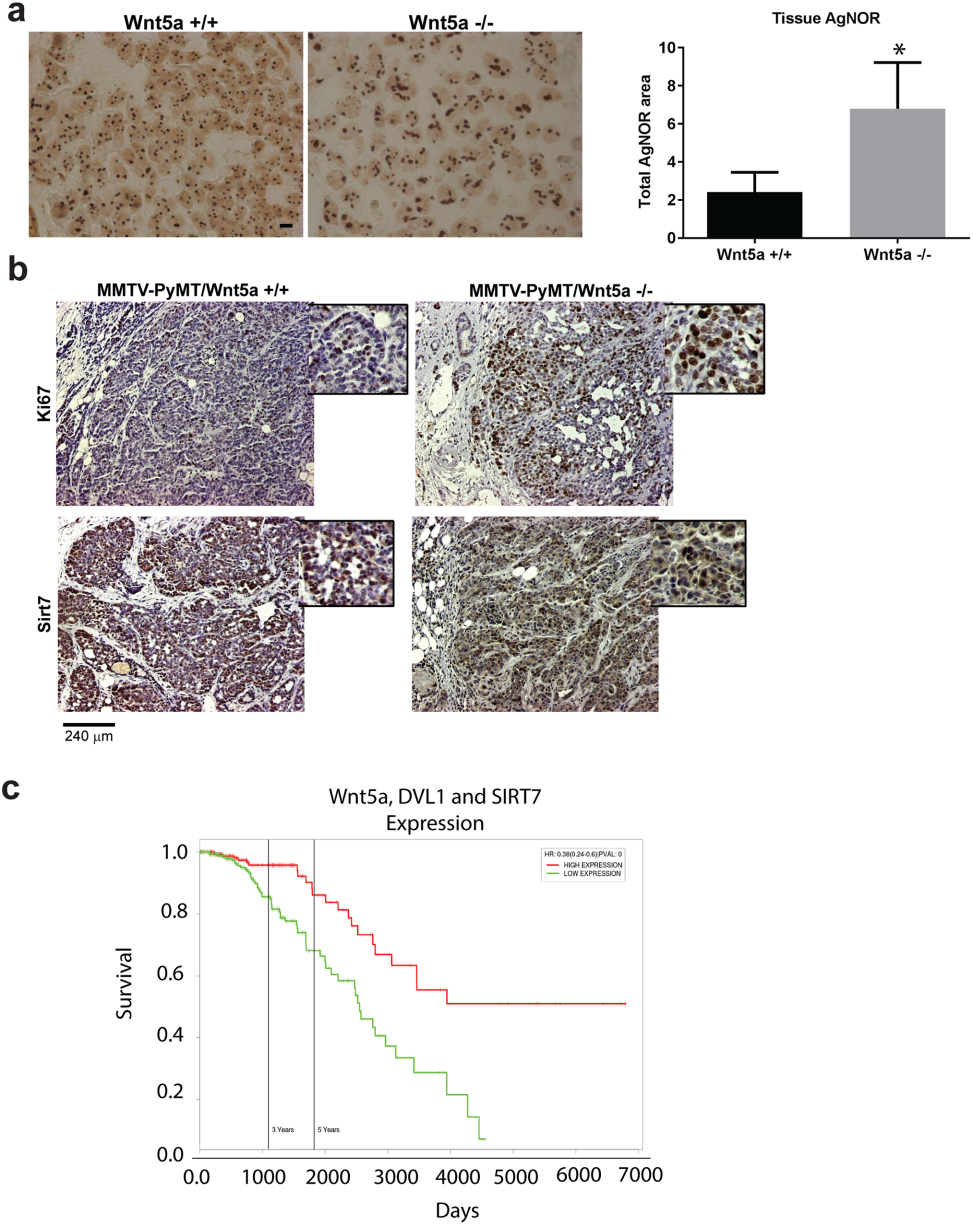
In mammary tumors, loss of Wnt5a expression led to increased nucleolar size, increased proliferation and changes in the distribution and expression level of the de-acetylase Sirt7. **(a)** AgNOR staining of tumors of MMTV-PyMT/Wnt5a^+/+^ and MMTV-PyMT/Wnt5a^-/-^. Scale bar = 10μm. Quantification of nucleoli staining showing a larger area in MMTV-PyMT/Wnt5a^+/+^ and MMTV-PyMT/Wnt5a^-/-^. Error bars indicate ± SD. *P < 0.05 (n = 3). (**b**) Immunostaining of breast tumors from MMTV-PyMT/Wnt5a^+/+^ and MMTV-PyMT/Wnt5a^-/-^ mice for Ki-67 and Sirt7. Scale bar = 240μm. (**c**) Survival curve showing that reduced expression of Wnt5a, DVL1 and SIRT7 correlates with lower survival of patients with breast cancer (TCGA data).

The ability of Wnt5a to inhibit rRNA synthesis and reduce nucleolar size suggests a possible mechanism by which it could constrain tumor growth *in vivo*. To address this possibility, we employed a mouse model of breast cancer with tumors driven by the MMTV-PyMT oncogene in wild-type and Wnt5a-null background [39]. As previously reported, Wnt5a-null mammary tumors exhibited increased Ki-67 expression levels compared to those derived from MMTV-PyMT/Wnt5a^+/+^ mice (Fig 5b) [39]. However, we also observed an increase in AgNOR staining of nucleolar areas in the Wnt5a^-/-^ tumors (Fig 5a). These effects suggest that the higher growth rate of Wnt5a^-/-^ tumors depends, at least in part, on elevated levels of rRNA synthesis. MMTV-PyMT/Wnt5a^-/-^ tumors also exhibited increased SIRT7 expression distributed throughout the tumor compared to those derived from MMTV-PyMT/Wnt5a^+/+^ mice (Fig 5b). Taken together, these data argue that anti-proliferative effects of Wnt5a are mediated through repression of rDNA transcription *in vivo*. In order to test the relevance of these findings to human disease, we analyzed publicly available expression data sets from cancer patients. We hypothesized that lower Wnt5a and DVL1 expression would correlate with a poorer prognosis and reduced patient survival. Survival curves generated using data from the Cancer Genome Atlas (TCGA) indeed corroborated this view (Fig 5c). Interestingly, higher levels of Wnt5a and DVL1 were correlated with higher survival rates irrespective of SIRT7 expression levels.

## Discussion

Ribosome availability is of central importance to the translational capacity of cells and their ability to synthesize biomass [1–3]. In both normal and cancerous cells, signals that attenuate the rate of rRNA synthesis have previously been shown to have a constraining effect on cell growth and proliferation [26]. Our observations in Wnt5a-deficient mammary tumors support this notion. While secreted growth factors are known to have positive effects on rRNA synthesis, and can contribute to oncogenesis, to our knowledge Wnt5a is the first example of a secreted factor that inhibits rRNA gene transcription [26]. In the context of human breast cancer, both epidemiological and functional data have implicated Wnt5a as a tumor suppressor and our data indicating that Wnt5a signals through DVL1 to inhibit rRNA synthesis provide a novel mechanism underlying that role [23–26]. Together with tumor suppressor proteins such as RB, p53, and other factors known to regulate nucleolar function [23], we suggest that loss of Wnt5a from breast tumor cells, or from their microenvironment, is one contributor to the enlarged nucleolar and elevated rRNA synthesis levels that are characteristic hallmarks of highly proliferative tumor cells. The molecular details of this Wnt5a-DVL1-SIRT7-rDNA signaling axis may reveal novel targets for drug development in the treatment of cancers whose rapid growth depends on deregulated ribosomal synthesis.

The roles of DVL proteins in Wnt signaling have mostly been associated with receptor-proximal aspects, reversible assembly of multi-molecular complexes, and/or interactions with components of the cytoskeleton [20,21,40,41]. However, previous studies have described DVL proteins within the nucleus, where they were implicated in activation of the TCF-responsive target genes of canonical Wnt/β-catenin signaling [19,42]. A large number of potential signaling consequences have been invoked for non-canonical signaling by Wnt5a in different contexts, many of which require DVL proteins as signaling intermediates [20,21,43]. Here we report that Wnt5a signaling leads to increased subcellular localization of DVL1 in the nucleolus, where it negatively regulates rRNA synthesis through loss of the de-acetylase SIRT7 from chromatin regions containing rDNA.

**Fig 6:**
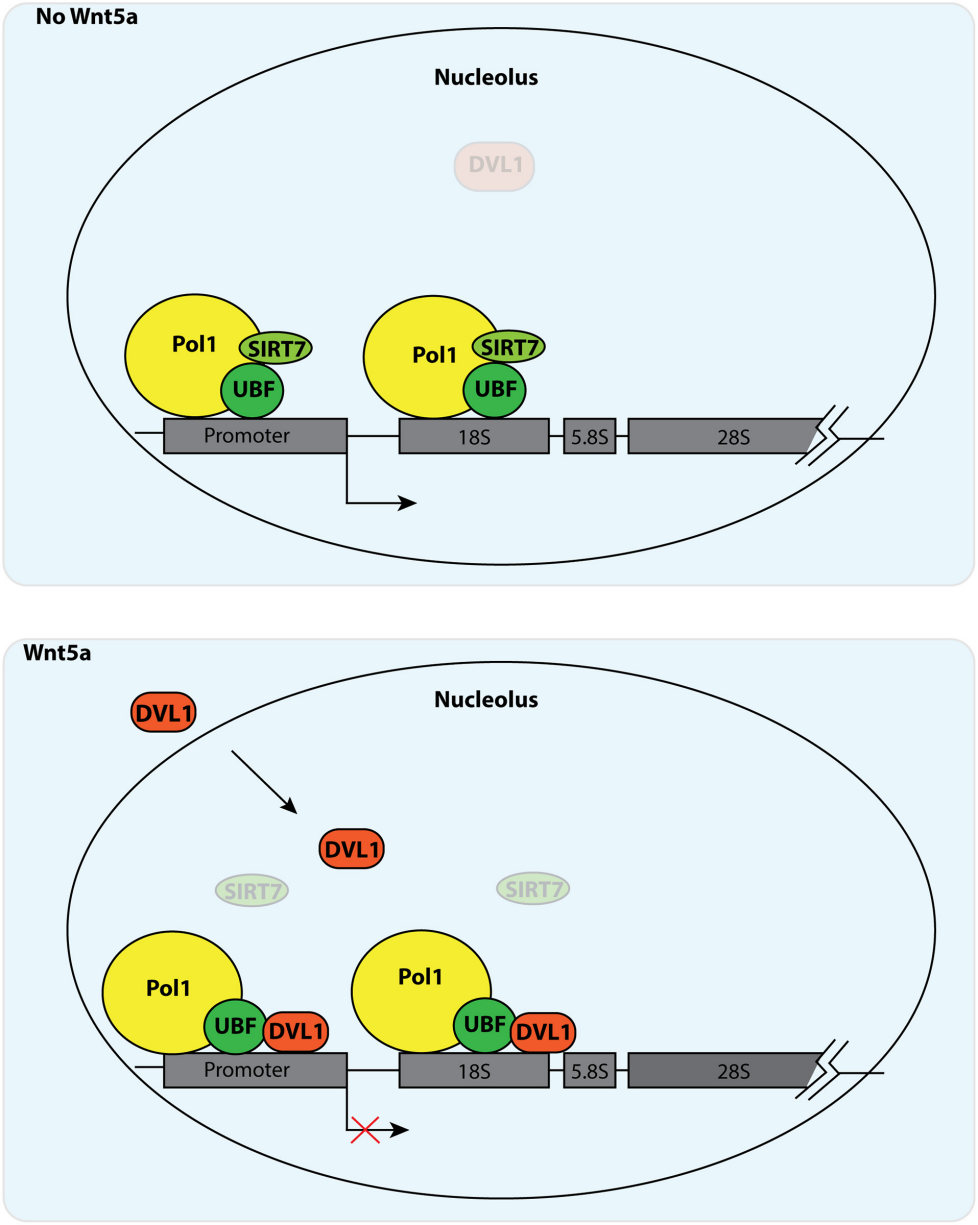
Model. In breast cancer cells, Wnt5a functions as tumor suppressor by inducing Pol I transcriptional repression through nucleolar accumulation of the downstream effector DVL1. In the presence of Wnt5a, DVL1 rapidly accumulates in the nucleolus where it binds to the rDNA transcription unit. DVL1 recruitment may be facilitated via a direct interaction with UBF, with concomitant loss of SIRT7 from active rRNA genes. These compositional changes in Pol I lead to transcriptional inhibition. In this model, Wnt5a exerts its tumor suppressive effects by inducing nucleolar accumulation of DVL1 and attenuating rRNA synthesis.

Our working model, which rationalizes the available data and explains the present observations, is that DVL1 and SIRT7 may bind to UBF in a mutually exclusive manner at active sites of rRNA synthesis (Fig 6). The DVL1-induced loss of SIRT7, which may be a direct or indirect effect, likely prevents the maintenance of the Pol I subunit PAF53 in a hypo-acetylated state, which is required for assembly of the transcription-competent Pol I machinery [13] (Fig 6). Considering that elevated levels of UBF are generally observed throughout the rDNA region upon transcription inhibition, we speculate that DVL1 may contribute to a mechanism that controls UBF deposition on rDNA chromatin for transcription repression, possibly modulating the number of active rDNA genes [7,37]. While further experiments will be needed to examine this mechanism in molecular detail, the present data reveal that extracellular signals can induce a novel pathway for the down-regulation of rDNA transcription. These observations provide further insight into the diverse cellular mechanisms and biological responses that can be regulated by Wnt signaling during development and disease.

## Materials and Methods

### Cell culture

MCF7, MDA-MB-231, and Rat2 cells were maintained in Dulbecco Modified Eagle’s Medium (DMEM) and BT549 in RPMI 1640 medium. All media contained 10% fetal bovine serum (FBS, Invitrogen) and 1% penicillin-streptomycin. Recombinant Wnt5a protein (R&D, #645WN) and Wnt3a (Peprotech, #315–20) were used at 200 ng/mL. Actinomycin D (Sigma-Aldrich A-9415) was added at 40 ng/mL or 1000 ng/mL MCF7 and BT549 cells expressing exogenous Wnt5a were generated by infection with the retrovirus vector LNCX containing Wnt5a cDNA, followed by selection of pooled colonies in G418 (Geneticin). Control cells were infected in parallel with the ‘empty’ vector. Rat2 fibroblasts transduced with FLAG-tagged DVL1 in an LNCX vector were constructed by Dr. José González-Sancho. Lentivirus particles expressing DVL1 shRNA (sc-35228-V), or non-silencing control shRNA were purchased from Santa Cruz Biotechnology Inc. and used to infect MCF7 and BT549 cells with selection in 5 μg/mL puromycin. ON-TARGETplus Human DVL1 (1855) siRNA—SMARTpool (L-004068-00-0005) and ON-TARGETplus Non-Targeting Pool (D-001810-10 from Dharmacon was used for nucleofection (Lonza Group Ltd, Switzerland) of MCF7 cells.

### Immunofluorescence

Cells were plated on glass cover slips at 50% confluency, one day before treatment with Wnt5a, vehicle, or Actinomycin D. Experiments were repeated at least three times. After treatment, cells were fixed in 4% formaldehyde for 15 min, quenched with 10 mM glycine, permeabilized with 0.1% or 0.3% Triton-X100 for 15 min, and blocked for 1 hour with 5% normal goat serum in PBS. In between these steps, cells were washed three times with PBS. Primary antibodies and their dilutions were as follows: Ki-67 (1:1000, RM-9106S1, Thermo Scientific); DVL1, (1:100, monoclonal 3F-12, Santa Cruz Biotech, SC-8025) and (1:100, polyclonal Biomol, DA4170); DVL2 (1:1000, Cell Signaling 3216); DVL3 (1:200, 4D3, Santa Cruz Biotech, SC- 8025); Fibrillarin, (1:1000, Abcam ab5821); UBF, (1:100, F-9, SC-13125, Santa Cruz Biotech.); β-catenin, (1:200, BD Transduction Labs, 610154), BrdU (1:200, Clone BU33, B2531, Sigma-Aldrich), FLAG (1:250, F4049, Sigma-Aldrich). Cells were stained for 60 min with primary antibodies diluted in PBS containing 0.1% BSA (PBSB). After two washes in PBSB, cells were incubated for 60 min with secondary antibodies diluted 1:1000 in PBSB. Secondary antibodies were: Alexa Fluor 488 goat anti-mouse, Alexa Fluor 546 goat anti-rabbit, Alexa Fluor 647 goat anti-rabbit (A11001, A11010 and A21244 Invitrogen Inc.). After antibody removal, cells were washed three times in PBSB, incubated with 1 mM TO-PRO (Invitrogen) in PBS or with DAPI to stain DNA, and washed twice more in PBS. Cover slips were mounted in SlowFade (Invitrogen Inc.) on microscope slides and visualized using either a Zeiss LSM510 or Zeiss LSM 710 confocal microscope. Cells were imaged using a 63X oil immersion lens, there were on average 20 cells per field. Each experiment was done in biological triplicate (n = 3) with each condition done in technical replicate (n = 2).

### Cell proliferation assays

Cells were seeded in 96-well plates at a density of 10^4^ cells per well in 100 μl of medium and their numbers evaluated over 4 successive days. For each 24 hour time point, 10 μl of 3-(4,5-dimethylthiazol-2-yl)-2,5-diphenyltetrazolium bromide (MTT) solution (Invitrogen; 5mg/ml in PBS) was added and the cells incubated for 4 hours at 37°C. After removing the medium, 75 μl dimethyl sulfoxide was added to each well to solubilize the precipitate, and absorbance at 540 nm was measured in a microplate reader (EL321e; Bio-Tek Instruments, Winooski, VT). Where indicated, the equivalent experiments were carried out in MCF7 cells subjected to shRNA-mediated silencing of DVL1.

### AgNOR staining on cells and mammary tumors

Silver staining of nucleoli was based on previously described AgNOR procedures [34,35]. Briefly, after fixation and rehydration, cells were stained with a freshly prepared AgNOR staining solution for 30 min. After staining, cells were rinsed twice in distilled water, treated with 5% sodium thiosulfate for 2–5 min, rinsed again, and mounted for bright field microscopy and image capture. ImageJ software was used to determine the total area of AgNOR staining in fields of 100 cells each [35]. Values were obtained from at least three experiments prior to analysis by Student’s *t*-test.

### Mouse mammary tumors

As previously described, mammary anlagen from mice carrying the MMTV-PyMT transgene in a Wnt5a^-/-^ or Wnt5a^+/+^ background were rescued from e18.5 day embryos by transplantation into cleared fat pads of 3-week old ICR/SCID hosts [39]. After expansion of the epithelium for one month, the tissue was transplanted again into ICR/SCID recipients and invasive adenocarcinomas of the two genotypes were recovered at 16 weeks. Paraffin-embedded fixed tissue from 3 tumors of each genotype was sectioned and processed for AgNOR staining as described and nucleolar area measured in fields of 100 nuclei as above. IHC staining of SIRT7, (1:150, 62748, Abcam,) and Ki-67 (1:150, RM-9106S1, Thermo Scientific) was done according to standard protocols.

### Semi-quantitative RT-PCR

MCF7 and BT549 cells were grown in 12-well dishes and, where indicated, treated with Wnt5a (200 ng/ml), sFRP1 (R&D, 1384-SF, 400 ng/ml) or a combination of both. In the case of double treatment, the cells were pre-treated with sFRP1 for one hour before addition of Wnt5a for 1 hour. RNA was isolated using an RNeasy Mini kit (Qiagen) according to the manufacturer’s protocol and treated with DNase1. cDNA was then synthesized with an iScript cDNA synthesis kit (Bio-Rad). The concentration of cDNA was determined by spectrophotometry, following which it was diluted in RNase-free water to a concentration of 10 ng/uL. Semi-quantitative RT-PCR was performed using 20 ng of cDNA template, a SYBR Green Fast-Mix PCR kit (Quanta Biosciences), and an MJ Research Opticon2 real-time PCR machine. The annealing temperature for 47S primers was 52.5°C and for all other primers was 55°C. All samples were run in triplicate. Primer sequences for 47S pre-rRNA were: forward, 5´-TGTCAGGCGTTCTCGTCTC-3´; reverse, 5´-GAGAGCACGACGTCACCAC-3´ (both from IDT, Inc.) [27]. Primers for DVL1 (QT01672944), β-actin (QT01680476) and Wnt5a (QT00025109) were from Qiagen (Quantitect). Fold changes in RNA levels were calculated using the delta-delta Ct method and analyzed by Student’s t-test [44].

### ChIP, qPCR and statistical analysis

ChIP assays were performed as previously described [33–34]. Formaldehyde cross-linked chromatin obtained from MCF7/Ctrl or MCF7/Wnt5a cells was subjected to immuno-precipitations with the autoimmune serum S57299 against Pol I [45] or with antibodies to DVL1, UBF, SIRT7, Histone H3K4me3 (HistoneH3K4me3, 39159, Active Motif), and non-specific rabbit IgGs (Abcam) as control. DNA-protein complexes were analyzed by qPCR with primers specific for the rDNA promoter (forward primer 5’- CCCGGGGGAGGTATATCTTT; reverse primer 5’- CCAACCTCTCCGACGACA), the 18S (Forward: 5’- CGGCTACCACATCCAAGGAA; reverse 5’- GCTGGAATTACCGCGGCT), the 28S rDNA repeat (Forward: 5’- CGACGACCCATTCGAACGTCT; reverse 5’- CTCTCCGGAATCGAACCCTGA) and the IGS (Forward: 5’- ATCTTGTTGTGCGGGAGTTC; reverse 5’- TTGTTCTGTCACTCGGTTGC). The qPCR analysis was performed as previously described and the results displayed as bars diagrams [45]. The values are presented as percentages of the input signal for each primer pair.

### Analysis of protein-protein interactions

Immunoprecipitation assays were performed as previously described [45]. Briefly, nuclear extracts of growing MCF7 cells or MCF7 cells expressing Wnt5a were incubated with the autoimmune serum S57299 against Pol I [45] or with antibodies against UBF, DVL1, or control non-specific rabbit immunoglobulins (IgGs). The antibodies were subsequently precipitated with Protein G Sepharose (Invitrogen). The beads were washed with 1X PBS supplemented with 1 mM PMSF, 0.2% NP-40 and then re-suspended in Laemmli buffer and heat denatured. Bound proteins were resolved by SDS-PAGE and analyzed on immunoblots for UBF, DVL1, SIRT7 and the largest Pol I subunit RPA194.

### Transcription assays

Active foci of Pol I-mediated transcription in MCF7 cells were revealed by FUrd incorporation as previously described [32].

### Immuno-electron microscopy

Sub-confluent cells were pelleted and fixed with 4% paraformaldehyde (Merck), dehydrated and embedded in Agar 100 resin (Agar Scientific Ltd). Thin sections were stained with 2% uranyl acetate and examined in a transmission electron microscope Tecnai G2 Spirit BioTwin (FEI Company) at 80 kV.

### Western blotting

Cells were lysed in RIPA buffer (50 mM Tris-HCl pH 7.5, 150 mM NaCl, 1 mM EDTA, 1% NP-40, 0.5% sodium deoxycholate, 0.1% SDS) supplemented with protease inhibitors (cOmplete cocktail, Roche). Protein extracts were boiled in gel sample buffer (Invitrogen), separated by SDS–PAGE under reducing conditions and transferred to nitrocellulose filters (Hybond, Amersham) by electro-blotting. Primary antibodies and dilutions used were DVL1 (3F-12, 1:200; SC-8025, Santa Cruz Biotech); DVL2 (1:1000, 3216, Cell Signaling); DVL3 (1:200, 4D3, Santa Cruz Biotech, SC-8025); Wnt5a (1:300; AF645, R&D); Pol I RPA194 (C1, sc-48385, Santa Cruz Biotech); UBF (1:500, F-9, Santa Cruz Biotech, SC-13125); SIRT7 (1:500; Abcam #62748); GAPDH (1:1000, 6C5, Abcam ab8245); β-actin (1:500, Abcam ab1801); Tubulin (1:500, Abcam, ab4074). Secondary antibodies include HRP conjugated, Mouse (GE, NA931V), Rabbit (NA931V, GE) or Rat (NA9340V), used at 1:10000. Blots were exposed using ECL using SuperSignal West Femto Maximum Sensitivity Substrate (Thermo Scientific, 34095) and visualized via BioRad ChemiDoc XRS imaging system.

### Survival analysis

Survival analysis was performed using the PROGgene V2 Prognostic Database (http://watson.compbio.iupui.edu/chirayu/proggene/database/?url=proggene) [46]. Each analysis used “breast cancer” as cancer type, “death” as survival measure, and bifurcated the gene expression at the median. The gene expression was taken from the TCGA database. The data were not adjusted for clinical status. The survival status was analyzed for expression levels of Wnt5a, DVL1 and SIRT7.

## Supporting Information

S1 Fig Wnt5a decreases nucleoli size via non-canonical Wnt signaling and reduces cell growth. **(a)** Immunofluorescence analysis of β-catenin (green) staining after treatment of BT549 cells for 3 hours with vehicle, Wnt3a (200 ng/mL) or Wnt5a (200 ng/mL). The inability of Wnt5a treatment to induce stabilization and nuclear translocation of β-catenin (green), unlike Wnt3a, indicates that it does not activate canonical Wnt/β-catenin signaling (n = 3). Scale bar = 10 μm. **(b)** Immunoblots of total lysates from BT549 cells expressing Wnt5a show that DVL2 is phosphorylated in a Wnt5a-dependent manner and GAPDH serves as a loading control [31]. (n = 3). **(c)** AgNOR staining of BT549 cells treated with vehicle, 200 ng/mL Wnt5a, or 1000 ng /mL Actinomycin D for 4 hours. Error bars indicate ± SD. Scale bar = 10 μm. Quantification at right shows that Wnt5a reduces the area of nucleoli. Image J software was used to compare the total area of AgNOR staining in equivalent numbers of cells. Error bars indicate ± SD. *P < 0.05; (n = 3). **(d)** AgNOR staining of BT549 and MCF7 cells stably expressing exogenous Wnt5a. Scale bar = 10 μm. Quantification shows that cells expressing exogenous Wnt5a have a reduced nucleolar area. Error bars indicate ± SD. (BT549, *P < 0.05) (n = 3). **(e)** MTT assay shows that BT549/Wnt5a cells proliferate more slowly than BT549 vector control cells. Viable cell numbers were determined by MTT assay over successive days. Results shown are from 3 independent experiments in which data points were obtained in quadruplicate. *P < 0.05 (n = 3).
(TIF)

S2 Fig DVL2 and DVL3 are excluded from nucleoli. **(a)** Immunofluorescence and confocal microscopy using antibodies to DVL1 (green) and Fibrillarin (red) merged with DNA (blue) in MCF7 cells and MCF7 cells stably expressing Wnt5a treated with ActD at 1000 ng/mL for 4 hours. **(b)** Immunofluorescence and confocal microscopy using antibodies to DVL2 (red) and UBF (green) in MCF7 cells and MCF7 cells stably expressing Wnt5a treated with vehicle or ActD at 40 ng/ml for 4 hours. Scale bar, 10μm (n = 3). Immunofluorescence and confocal microscopy using antibodies to DVL3 (green) and Fibrillarin (red) in MCF7 cells and MCF7 cells stably expressing Wnt5a treated with vehicle or ActD at 40 ng/ml for 4 hours. Scale bar, 10μm (n = 3).
(TIF)

S3 Fig Nucleolar localization of DVL1. **(a)** Immunofluorescence with rabbit polyclonal antibody for DVL1 (red) merged with DNA (blue) in MCF7 and MDA-MB-231 breast cancer cells. Scale bar, 10 μm. (n = 3). **(b)** Exogenous DVL1 ectopically expressed in Rat2 cells localizes to the nucleolus. Immunofluorescence of DVL1 (green) and Fibrillarin (red) and their co-localization (yellow, right) in Rat2 cells transduced with a FLAG-tagged DVL1 retrovirus (Rat2/DVL1) or control vector (Rat2/Ctrl). Exogenous DVL1 in Rat/DVL1 fibroblast cells was also detected with FLAG antibody (green) and co-localized with Fibrillarin (red). Scale bar, 10 μm. (n = 3). **(c)** Immuno-gold transmission electron micrographs of MCF7 cells stably expressing Wnt5a (MCF7/Wnt5a) and MDA-MB-231 cell nuclei, showing DVL1 within nucleoli (arrows). Small arrowheads in the upper panel point to the nuclear envelope. Scale bar, 500nm. All experiments were performed at least three times (n = 3), except immuno-EM which was performed twice. **(d**) Immunoblots of lysates of MCF7 cells stably expressing Wnt5a (MCF7/Wnt5a) show unaltered levels of DVL1, SIRT7 and UBF expression with respect to control MCF7 cells (Ctrl) not expressing Wnt5a. GAPDH and Actin were used as loading controls. (n = 3).
(TIF)

S4 Fig Knockdown of endogenous DVL1 does not affect DVL2 or DVL3 levels. **(a)** Western blots showing specific reduction of DVL1 protein levels, but no change in DVL2 or DVL3, in BT549 and MCF7 cells transduced with DVL1 shRNA (shDVL1) compared to non-silencing shRNA (Ctrl). Tubulin and GAPDH served as loading controls (n = 3). **(b)** Nucleofection of MCF7 cells with siRNA oligonucleotides reduces DVL1 RNA levels (top) and causes up-regulation of 47S pre-rRNA expression (bottom), confirming results obtained with shRNA-mediated silencing of DVL1 in Fig 4b. Error bars indicate ± SD (n = 3).
(TIF)
