# Supplementary figures and images for: Wnt5a Signals through DVL1 to Repress Ribosomal DNA Transcription by RNA Polymerase I
Source: PLoS Genet. 2016 Aug 8;12(8):e1006217. doi: 10.1371/journal.pgen.1006217 (PMC4976976; doi:10.1371/journal.pgen.1006217)

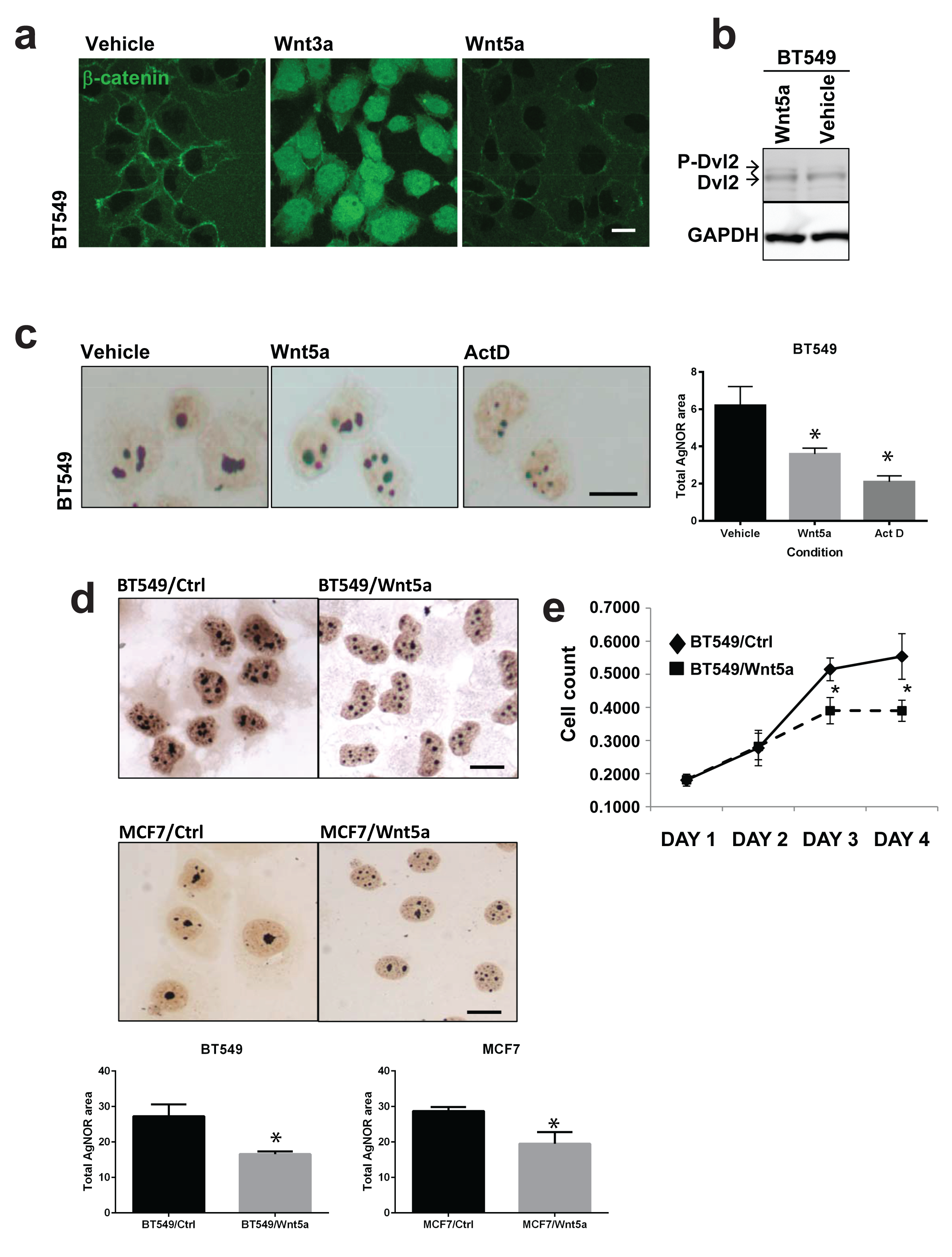

Supplement: S1 Fig — (a) Immunofluorescence analysis of β-catenin (green) staining after treatment of BT549 cells for 3 hours with vehicle, Wnt3a (200 ng/mL) or Wnt5a (200 ng/mL). The inability of Wnt5a treatment to induce stabilization and nuclear translocation of β-catenin (green), unlike Wnt3a, indicates that it does not activate canonical Wnt/β-catenin signaling (n = 3). Scale bar = 10 μm. (b) Immunoblots of total lysates from BT549 cells expressing Wnt5a show that DVL2 is phosphorylated in a Wnt5a-dependent manner and GAPDH serves as a loading control [31]. (n = 3). (c) AgNOR staining of BT549 cells treated with vehicle, 200 ng/mL Wnt5a, or 1000 ng /mL Actinomycin D for 4 hours. Error bars indicate ± SD. Scale bar = 10 μm. Quantification at right shows that Wnt5a reduces the area of nucleoli. Image J software was used to compare the total area of AgNOR staining in equivalent numbers of cells. Error bars indicate ± SD. *P < 0.05; (n = 3). (d) AgNOR staining of BT549 and MCF7 cells stably expressing exogenous Wnt5a. Scale bar = 10 μm. Quantification shows that cells expressing exogenous Wnt5a have a reduced nucleolar area. Error bars indicate ± SD. (BT549, *P < 0.05) (n = 3). (e) MTT assay shows that BT549/Wnt5a cells proliferate more slowly than BT549 vector control cells. Viable cell numbers were determined by MTT assay over successive days. Results shown are from 3 independent experiments in which data points were obtained in quadruplicate. *P < 0.05 (n = 3). (TIF) [file pgen.1006217.s001.tif]

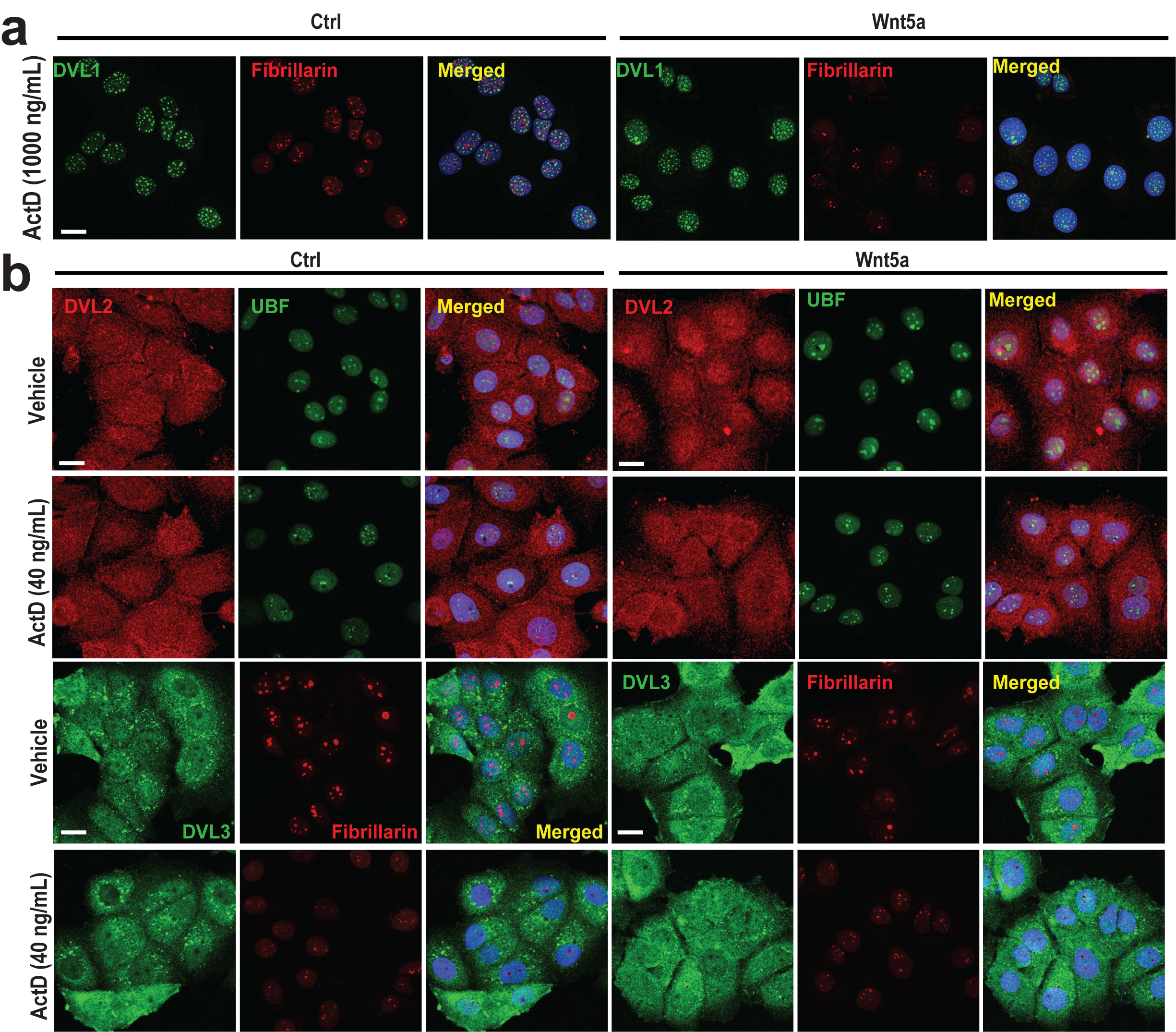

Supplement: S2 Fig — (a) Immunofluorescence and confocal microscopy using antibodies to DVL1 (green) and Fibrillarin (red) merged with DNA (blue) in MCF7 cells and MCF7 cells stably expressing Wnt5a treated with ActD at 1000 ng/mL for 4 hours. (b) Immunofluorescence and confocal microscopy using antibodies to DVL2 (red) and UBF (green) in MCF7 cells and MCF7 cells stably expressing Wnt5a treated with vehicle or ActD at 40 ng/ml for 4 hours. Scale bar, 10μm (n = 3). Immunofluorescence and confocal microscopy using antibodies to DVL3 (green) and Fibrillarin (red) in MCF7 cells and MCF7 cells stably expressing Wnt5a treated with vehicle or ActD at 40 ng/ml for 4 hours. Scale bar, 10μm (n = 3). (TIF) [file pgen.1006217.s002.tif]

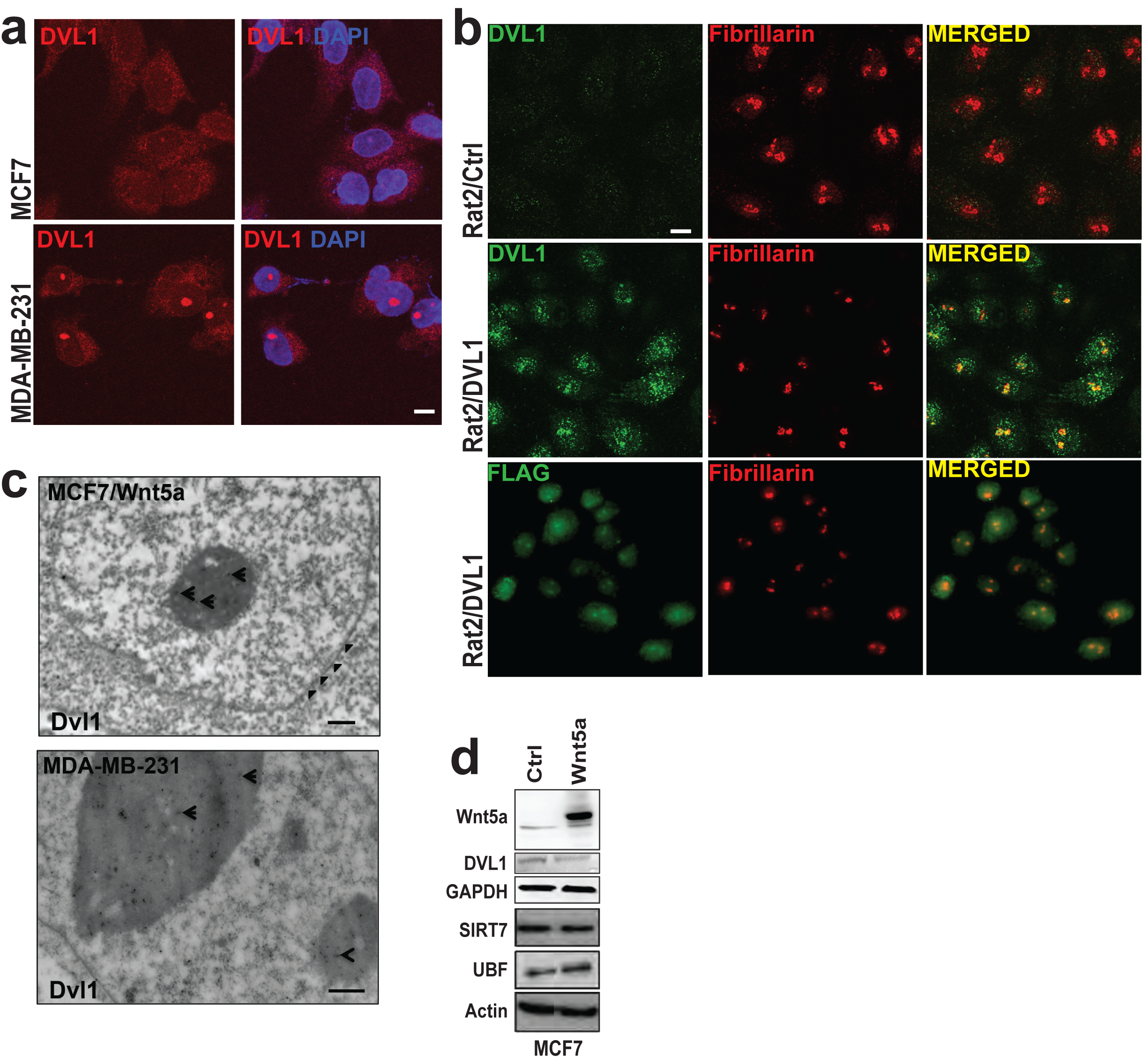

Supplement: S3 Fig — (a) Immunofluorescence with rabbit polyclonal antibody for DVL1 (red) merged with DNA (blue) in MCF7 and MDA-MB-231 breast cancer cells. Scale bar, 10 μm. (n = 3). (b) Exogenous DVL1 ectopically expressed in Rat2 cells localizes to the nucleolus. Immunofluorescence of DVL1 (green) and Fibrillarin (red) and their co-localization (yellow, right) in Rat2 cells transduced with a FLAG-tagged DVL1 retrovirus (Rat2/DVL1) or control vector (Rat2/Ctrl). Exogenous DVL1 in Rat/DVL1 fibroblast cells was also detected with FLAG antibody (green) and co-localized with Fibrillarin (red). Scale bar, 10 μm. (n = 3). (c) Immuno-gold transmission electron micrographs of MCF7 cells stably expressing Wnt5a (MCF7/Wnt5a) and MDA-MB-231 cell nuclei, showing DVL1 within nucleoli (arrows). Small arrowheads in the upper panel point to the nuclear envelope. Scale bar, 500nm. All experiments were performed at least three times (n = 3), except immuno-EM which was performed twice. (d) Immunoblots of lysates of MCF7 cells stably expressing Wnt5a (MCF7/Wnt5a) show unaltered levels of DVL1, SIRT7 and UBF expression with respect to control MCF7 cells (Ctrl) not expressing Wnt5a. GAPDH and Actin were used as loading controls. (n = 3). (TIF) [file pgen.1006217.s003.tif]

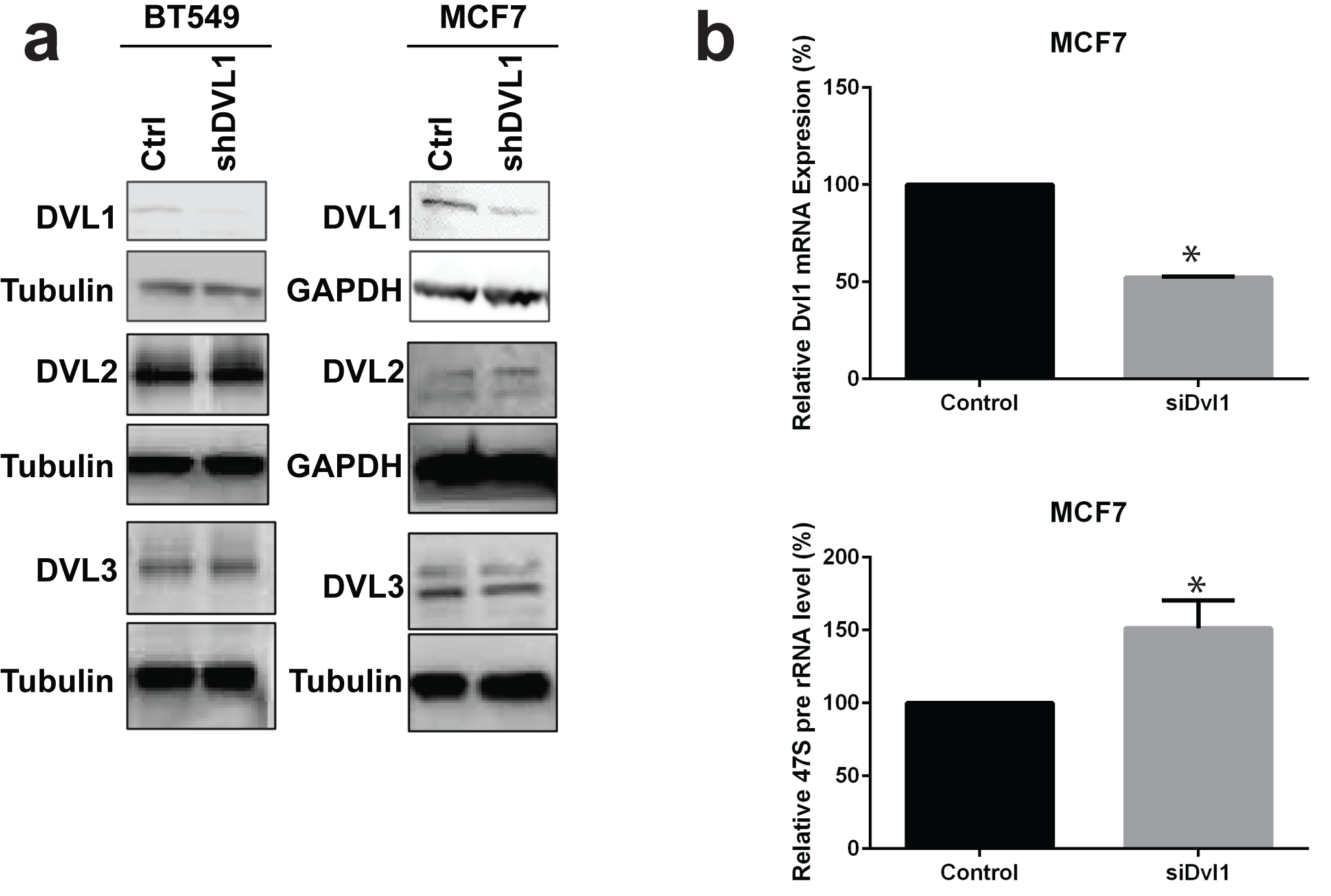

Supplement: S4 Fig — (a) Western blots showing specific reduction of DVL1 protein levels, but no change in DVL2 or DVL3, in BT549 and MCF7 cells transduced with DVL1 shRNA (shDVL1) compared to non-silencing shRNA (Ctrl). Tubulin and GAPDH served as loading controls (n = 3). (b) Nucleofection of MCF7 cells with siRNA oligonucleotides reduces DVL1 RNA levels (top) and causes up-regulation of 47S pre-rRNA expression (bottom), confirming results obtained with shRNA-mediated silencing of DVL1 in Fig 4b. Error bars indicate ± SD (n = 3). (TIF) [file pgen.1006217.s004.tif]
